# Associated factors of willingness to undergo routine chlamydia trachomatis screening among hospital-based patients in Shenzhen, China: a cross-sectional study

**DOI:** 10.1186/s12889-020-09828-6

**Published:** 2020-11-16

**Authors:** Rongxing Weng, Fuchang Hong, Chunlai Zhang, Lizhang Wen, Xiangsheng Chen, Yumao Cai

**Affiliations:** 1 Department of STD Control and Prevention, Shenzhen Center for Chronic Disease Control, No. 2021, Buxin Road, Luohu District, Shenzhen City, 518020 Guangdong Province China; 2 Peking Union Medical College Institute of Dermatology, Chinese Academy of Medical Science and Peking Union Medical College, Nanjing, 210042 China; 3 National Center for Sexually Transmitted Disease Control, Nanjing, China

**Keywords:** *Chlamydia trachomatis*, Routine screening, Willingness, Associated factors

## Abstract

**Background:**

Chlamydia trachomatis (CT) is a common sexually transmitted infection (STI) with significant morbidity. The study aimed to explore the willingness to undergo routine CT screening and its associated factors among hospital-based patients in Shenzhen.

**Methods:**

We used data from the Shenzhen Gonorrhea and Chlamydia Intervention Programme. Participants were recruited with a stratified purposeful sampling design from 1 April 2018 to 16 May 2018. A structured questionnaire was used to obtain data on baseline characteristics and CT-related participant information.

**Results:**

Of the 16,546 participants, 64.79% were women, with a mean age of 31.85 ± 7.31 of all participants. Of the participants, 88.78% were willing to undergo routine CT screening. According to multivariate logistic regression analyses, willingness to undergo routine CT screening was associated with the following (*P* < 0.05): being a woman (AOR = 1.53, 95% CI = 1.34–1.75), one year or more residency in Shenzhen (AOR = 1.64, 95% CI = 1.37–1.95), any secondary education (AOR = 2.46, 95% CI = 1.92–3.15), monthly income ≥ RMB 10,000 (AOR = 1.24, 95% CI = 1.01–1.51), having forgotten CT diagnosis history (AOR = 1.42, 95% CI = 1.12–1.79), without current STI-related symptoms (AOR = 1.24, 95% CI = 1.10–1.41), and having correct understanding of the sequelae of CT infection (AOR = 1.68, 95% CI = 1.39–2.03).

**Conclusion:**

This study reported high willingness to undergo routine CT screening among hospital-based patients in Shenzhen, and provided evidence for the promotion and the implementation of strategies and recommendations on routine CT screening in China.

**Supplementary Information:**

The online version contains supplementary material available at 10.1186/s12889-020-09828-6.

## Background

*Chlamydia trachomatis* (CT) is a common sexually transmitted infection (STI) with significant morbidity. According to the World Health Organization (WHO), the global estimate of new CT cases was 127.2 million in 2016 [1]. Untreated CT infection can cause several complications, such as pelvic inflammatory disease (PID), tubal infertility, ectopic pregnancy, and chronic pelvic pain in women [2, 3]; and epididymitis, nongonococcal urethritis, and infertility in men [4, 5]. About 85% of CT infections in women and men are asymptomatic, which indicates that CT screening is important in detecting these asymptomatic cases [6].

CT screening among young sexually active adults is recommended in many developed countries, including the USA, Denmark, Australia, the UK, Norway, and Sweden [7–9]. A nationwide scheme called the National Chlamydia Screening Programme (NCSP) was put forward in the UK [10], and all sexually active men and women aged under 25 years were targeted for annual chlamydia screening through various clinical and nonclinical settings. Similarly, the Shenzhen Gonorrhea and Chlamydia Intervention Programme (SGCIP), a pilot project of a national programme in China (the China Chlamydia Intervention Programme), was launched in 2017, and it was the first city-level CT screening programme in China [11]. However, there are no policy strategies for routine CT screening in China. Besides, adults aged 25 years or older should not be neglected in CT screening in China. A study in China reported a high prevalence (9.09%) of CT infection among hospital-based patients aged > 25 years [11]. It is known that CT screening in the population with CT prevalence > 3% is cost-effective [12].

The CT screening programme is now an important part of the public health system. However, little is known about the acceptability of routine CT screening in the Chinese context, and it may be sensitive to undergo STI screening because of social stigma and invasive specimen collection [13]. Understanding which characteristics are associated with the willingness to undergo routine CT screening could inform health authorities in developing interventions and policy recommendations. This study, as part of the pilot project in China (SGCIP), aimed to explore the willingness to undergo routine CT screening, and to identify associated factors among hospital-based patients in Shenzhen.

## Methods

### Setting and methods of participant selection

We used data from a baseline study in the Shenzhen Gonorrhea and Chlamydia Intervention Programme. In this study, a stratified purposeful sampling design was used to identify study sites to include the study on the basis of the highest number of reported CT cases in 2017. Initially, we selected the administrative districts according to the number of reported CT cases. As hospitals in one district declined to participate in the study and the number of reported CT cases in three other districts was negligible, six of ten administrative districts in Shenzhen were included in the study. Second, the top four hospitals in each district for the number of reported CT cases were selected except for one district, with only two hospitals. A total of 22 hospitals were selected in this stage. Third, the top three departments for the number of reported CT cases in Shenzhen were identified (departments of obstetrics and gynecology, urology, and dermatology and venereology) and selected as the study sites in each hospital. From 1 April 2018 to 16 May 2018, the first 15 eligible patients per day in each department in the selected hospitals were asked to participate in a questionnaire interview after confirming the inclusion criteria of the participant and obtaining written informed consent. Inclusion criteria for participants comprised (1) age between 18 and 49 years, (2) having ever engaged in sexual intercourse, and (3) having had no antibiotic use in the last 2 weeks.

### Questionnaire survey

#### Main process

Training about how to conduct the questionnaire interview according to the study protocols was provided for the investigation staff in each included hospital. After the inclusion criteria of the participant were confirmed, and informed consent was obtained, a structured questionnaire was used to obtain data on the baseline characteristics, CT knowledge, and screening willingness of the participants (details of the questionnaire given in Additional file 1).

### Baseline characteristics and CT-related participant information

The following variables of baseline characteristics were gathered: gender, age, marital status, separation, type and length of residency, education level, health insurance, monthly income, and sexual orientation. Variables describing CT-related information were mainly on chlamydia testing and diagnosis history, STI-related symptoms, having a new sexual partner or multiple sex partners in the last 3 months, and CT screening willingness.

### Questions about willingness to undergo routine CT screening, chlamydia related-knowledge, and definition of Shenzhen *Hukou* and length of residency

The question “are you willing to undergo routine CT screening once a year?” was intended to obtain the willingness to undergo routine CT screening from participants with two response options (“no” and “yes”). Two questions (Knowledge Q1 and Q2) were related to chlamydia infection knowledge, with 4 items in each question. For Knowledge Q1 “what do you know about genital *Chlamydia trachoma* infections?”, four response options were provided: (a) “never heard of it”, (b) “a kind of infectious disease”, (c) “a kind of genital tract infection”, and (d) “a kind of sexually transmitted disease”. Response options were coded as follows: (a) “lack of understanding”, and (b), (c), and (d) “Correct understanding”, resulting in two knowledge levels. For Knowledge Q2 “what do you know about the dangers of genital *chlamydia trachomatis* infections on the human body?”, four response options were provided: (a) “no dangers”, (b) “may affect sexual life”, (c) “may affect fertility”, and (d) “know nothing about it”. Response options were coded as follows: (a) or (d) “lack of understanding”, and (b) or (c) “correct understanding”, resulting in two levels of knowledge. Definition of the Shenzhen *Hukou*: *Hukou* system is a household registration system in China that is used to divide the Chinese population into agricultural and non-agricultural *Hukou* holders, which helps the government regulate population flows [14]. People with Shenzhen *Hukou* are those who have registered their permanent residence in Shenzhen. Length of residency is the length for which the participants have lived in the city of Shenzhen.

### Statistical analysis

All data from the questionnaires were double entered into the computer using Epi Data software (Epi Data for Windows; The Epi Data Association Odense, Denmark) to establish a dataset. Mean ± standard deviation (SD) was used to describe the distribution of continuous variables, and frequency (%) was used to describe categorical variables. Crude odds ratios (OR) and their 95% confidence interval (CI) were calculated using univariate logistic regression analysis, and variables with *P* < 0.2 were included in multivariate logistic regression analysis using a forward stepwise procedure to obtain adjusted odds ratios (AOR) and their 95% CIs. Variance inflation factor (VIF) was calculated to assess the collinearity of independent variables, and an independent variable with VIF > 5 was considered as highly collinear. We adopted a multivariate logistic regression model, defining screening willingness as a dependent variable, and gender, age, marital status, separation, Shenzhen *Hukou*, length of residency, education, insurance, monthly income, sexual orientation, chlamydia testing and diagnosis history, STI-related symptoms, and Knowledge Q1 and Q2 as independent variables. All data analysis was performed on Statistical Package for Social Sciences (SPSS) version 21.0 software (SPSS Inc., Chicago, IL, USA). All tests were two-tailed, and *P* < 0.05 was considered as statistically significant.

## Results

### Baseline and other participant characteristics

Baseline and other characteristics of participants are summarized in Table 1. Of the participants, 723 who did not complete the question about willingness to undergo routine CT screening were excluded, leaving 16,546 participants (95.81% of the participants) in final data analysis. Of the 16,546 participants, 64.79% were women, with a mean age of 31.85 ± 7.31 for all participants. Of the participants, 85.67% were older than 24 years old, and about three-quarters (73.73%) were married. Around three-fourths (72.13%) had not registered Shenzhen *Hukou*, and 87.93% had lived in Shenzhen 1 year or more. With regard to participant behaviors, only 8.79% had been tested for chlamydia infections, 6.27% had been diagnosed, and 38.02% had had a new sexual partner or multiple sex partners in the last 3 months.

**Table 1:** Baseline characteristics, knowledge and screening willingness of participants

Variables	Frequency (*%*)^a^
Gender (*n* = 16,513)	
Men	5814 (35.21%)
Women	10,699 (64.79%)
Age groups (*n* = 16,457)	
≤ 24	2359 (14.33%)
> 24	14,098 (85.67%)
Marital status (*n* = 16,473)	
Single/Divorced/Widowed	4327 (26.27%)
Married	12,146 (73.73%)
Living separate and apart (*n* = 13,219)	
Yes	1807 (13.67%)
No	11,412 (86.33%)
Shenzhen *Hukou* (*n* = 16,248)	
No	11,720 (72.13%)
Yes	4528 (27.87%)
Length of residency (*n* = 16,325)	
< 1 year	1971 (12.07%)
≥ 1 year	14,354 (87.93%)
Education level (*n* = 16,381)	
Primary school	4692 (28.64%)
Any secondary	11,689 (71.36%)
Monthly income (RMB) (*n* = 15,290)	
0–4999	5361 (35.06%)
5000-9999	6942 (45.40%)
10,000-	2987 (19.54%)
Health insurance (*n* = 16,394)	
No	5788 (35.31%)
Yes	10,606 (64.69%)
Sexual orientation (*n* = 16,271)	
Homosexuality/bisexuality	300 (1.84%)
Heterosexuality	15,971 (98.16%)
Ever CT tested (*n* = 16,229)	
No	14,803 (91.21%)
Yes	1426 (8.79%)
Ever CT diagnosed (*n* = 16,417)	
No	13,835 (84.27%)
Yes	1029 (6.27%)
Forgot	1553 (9.46%)
Current STI-related symptoms (*n* = 16,326)	
Yes	9659 (59.16%)
No	6667 (40.84%)
Having a new sexual partner or multiple sex partners in last 3 months (*n* = 16,319)	
Yes	6205 (38.02%)
No	10,114 (61.98%)
Knowledge Q1 (*n* = 16,400)	
Lack of understanding	12,085 (73.69%)
Correct understanding	4315 (26.31%)
Knowledge Q2 (*n* = 16,508)	
Lack of understanding	12,369 (74.93%)
Correct understanding	4139 (25.07%)
Routine CT screening willingness (*n* = 16,546)	
Unwilling	1856 (11.22%)
Willing	14,690 (88.78%)

*Abbreviations*: *CT* Chlamydia trachomatis, *STI* Sexually transmitted infections

^a^%: Constituent ratio

### CT-related knowledge and screening willingness of participants

CT-related knowledge and screening willingness of the participants are also shown in Table 1. Most participants (88.78%) were willing to undergo routine CT screening. Around three-quarters of the participants lacked an understanding of chlamydia infections (Knowledge Q1 = 73.69%; Q2 = 74.93%).

### Factors associated with willingness to undergo routine CT screening

In univariate analyses, 15 variables were associated with screening willingness at *P* < 0.20 (Table 2). As shown in Table 3, the VIF value among all independent variables was lower than 5, so no collinearity was detected. Results from the multivariate logistic regression model suggested that the following factors were significantly associated with screening willingness (*P* < 0.05): being a woman (AOR = 1.53, 95% CI = 1.34–1.75), 1 year or more residency in Shenzhen (AOR = 1.64, 95% CI = 1.37–1.95), any secondary education (AOR = 2.46, 95% CI = 1.92–3.15), monthly income ≥ RMB 10,000 (AOR = 1.24, 95% CI = 1.01–1.51), having forgotten CT diagnosis history (AOR = 1.42, 95% CI = 1.12–1.79), without current STI-related symptoms (AOR = 1.24, 95% CI = 1.10–1.41), and having correct understanding of the sequelae of CT infection (AOR = 1.68, 95% CI = 1.39–2.03).

**Table 2:** Multivariable logistic regression analysis of factors associated with willingness to undergo routine CT screening

Variables	Crude OR (95% CI)	*P* Values	Adjusted OR (95% CI)	*P* Values
Gender				
Men	Reference		Reference	
Women	1.42 (1.29,1.57)	< 0.001^**^	1.53 (1.34,1.75)	< 0.001^**^
Age groups				
≤ 24	Reference		Reference	
> 24	1.23 (1.08,1.40)	0.002^*^	1.11 (0.87,1.40)	0.401
Marital status				
Single/Divorced/Widowed	Reference		Reference	
Married	1.18 (1.06,1.32)	0.002^*^	1.10 (0.89,1.35)	0.368
Living separate and apart				
Yes	Reference		Reference	
No	1.16 (1.00,1.35)	0.057	0.97 (0.81,1.15)	0.732
Shenzhen *Hukou*				
No	Reference		Reference	
Yes	1.36 (1.21,1.52)	< 0.001^**^	1.12 (0.96,1.31)	0.152
Length of residency				
< 1 year	Reference		Reference	
≥ 1 year	1.81 (1.60,2.06)	< 0.001^**^	1.64 (1.37,1.95)	< 0.001^**^
Education level				
Primary school	Reference		Reference	
Any secondary \	2.89 (2.37,3.52)	< 0.001^**^	2.46 (1.92,3.15)	< 0.001^**^
Monthly income (RMB)				
0–4999	Reference		Reference	
5000-9999	1.09 (0.97,1.21)	0.142	0.96 (0.83,1.11)	0.566
10,000-	1.44 (1.24,1.68)	< 0.001^**^	1.24 (1.01,1.51)	0.036^*^
Health insurance				
No	Reference		Reference	
Yes	1.14 (1.03,1.26)	0.012^*^	0.92 (0.80,1.05)	0.214
Sexual orientation				
Homosexuality/bisexuality	Reference		Reference	
Heterosexuality	0.69 (0.45,1.05)	0.081	0.75 (0.42,1.34)	0.337
Ever CT tested				
No	Reference		Reference	
Yes	1.84 (1.48,2.28)	< 0.001^**^	1.16 (0.85,1.57)	0.355
Ever CT diagnosed				
No	Reference		Reference	
Yes	2.34 (1.78, 3.07)	< 0.001^**^	1.44 (0.97,2.14)	0.068
Forgot	1.61 (1.33, 1.95)	< 0.001^**^	1.42 (1.12,1.79)	0.004^*^
Current STI-related symptoms				
Yes	Reference		Reference	
No	1.18 (1.07,1.31)	0.001^*^	1.24 (1.10,1.41)	0.001^*^
Having a new sexual partner or multiple sex partners in last 3 months				
Yes	Reference			
No	0.98 (0.88,1.08)	0.639		
Knowledge Q1				
Lack of understanding	Reference		Reference	
Correct understanding	1.43 (1.27,1.61)	< 0.001^**^	1.01 (0.85,1.21)	0.902
Knowledge Q2				
Lack of understanding	Reference		Reference	
Correct understanding	1.85 (1.63,2.11)	< 0.001^**^	1.68 (1.39,2.03)	< 0.001^**^

*Abbreviations*: *CI* Confidence interval, *CT* Chlamydia trachomatis, *OR* Odds ratio, *STI* Sexually transmitted infections

^*^P < 0.05, ^**^P < 0.001

**Table 3:** The VIF value of all independent variables

Variables	VIF
Gender	1.131
Age groups	1.219
Marital status	1.205
Living separate and apart	1.037
Shenzhen *Hukou*	1.224
Length of residency	1.123
Education level	1.075
Monthly income (RMB)	1.250
Health insurance	1.156
Sexual orientation	1.004
Ever CT tested	1.293
Ever CT diagnosed	1.210
Current STI-related symptoms	1.033
Knowledge Q1	1.508
Knowledge Q2	1.497

*Abbreviations*: *CT* Chlamydia trachomatis, *STI* Sexually transmitted infections, *VIF* Variance inflation factor

## Discussion

Rarely have studies reported on the screening willingness for chlamydia infections in China, and this is the first hospital-based and multisite survey to report a willingness to undergo routine CT screening in China. Our study indicated a high willingness in our study population (88.8%), and that was reported in several previously conducted studies in China and other countries, having great range. A regional study in China reported that willingness to undergo STI tests among rural-to-urban migrants was 60.1% in Beijing and Nanjing [15]. A study in the United States showed that willingness to have STI tests among adolescents was 92.1% [16]. It was reported that the willingness to have CT tests was 66.3% in the Netherlands [17]. In addition, most participants in our study (91.2%) had not been tested before, suggesting that the expansion of CT screening should be considered in future interventions in those patients without a CT test history. Our findings have proven the data in support of routine CT screening projects, and provide evidence for the promotion and implementation of strategies and recommendations on routine CT screening in China to detect more CT cases.

The present study showed that women were more willing to undergo routine CT screening than men, which is consistent with the findings of a previous study [17]. It was reported that the main motivation of men on CT screening was health reasons, and men who were not willing to have CT screening may have fewer health concerns and underestimate their risk, so they in particular need to obtain CT information [17–19]. However, the result in this study was inconsistent with that in China [15].

The significantly higher willingness to undergo routine CT screening among participants with a higher education level compared to participants with a primary school education level indicated that the population with a low education level had the lowest willingness in this study, which was consistent with past studies [15, 20–22]. However, a lower education level is not the only factor. In the current study, participants with a longer length of residency in Shenzhen and higher monthly income were more willing to undergo routine CT screening. A possible reason for these observations is as follows: there is a large scale of rural-to-urban migrants in China for numerous economic opportunities, and this population was found to have limited education, shorter length of residency, low income to pay for relatively expensive health services, restricted reproductive health information, and limited access to health services, and to be more vulnerable to STIs [23, 24]. Therefore, rural-to-urban migrants, especially migrants with low levels of education, shorter length of residency and lower monthly income should be considered more in health education strategies. This study indicated that the topic of willingness among rural-to-urban migrants should be further considered in future research.

This study also showed that participants with more knowledge about the dangers of CT were more willing to undergo routine CT screening, which was in accordance with findings in other studies [21, 22, 25]. If individuals were informed that chlamydia is a serious condition and common, and could be asymptomatic, they would be more willing to undergo CT screening [25]. Health education interventions could be implemented through doctors, health leaflets, TV ads, magazines, and schools [25].

The current study reported that more than seven-tenths of the participants lacked an understanding of chlamydia infections (Knowledge Q1 = 73.69%; Q2 = 74.93%). It is not surprising that many people in the community or going to the clinics are entirely uninformed about urogenital infections of chlamydia (including the danger of infection) in China and in many other countries or areas. Even some health workers working in obstetrics and gynecology do not care about the dangers of CT infections. People sometimes complain about discomfort in the genital tract, but are not aware of any specific infections, including chlamydia. Besides, most of chlamydia infections are asymptomatic. Knowledge is sometimes associated with testing behaviors, but sometimes it is not. For the current survey, participants were told about what the test meant and what the test did for, although we did not pass on intensive education about chlamydia before the questionnaire survey. Therefore, the knowledge and willingness results in our survey reflected real-world situations, and they can be used as background for designing intervention programmes. The impact of intensive or innovative education about chlamydia and its dangers on the willingness to undergo testing or the testing uptake may be a topic for future study.

The current study also found that participants without STI-related symptoms were more willing to undergo routine CT screening, which was inconsistent with a previous systematic review [25]. Symptomatic participants may be affected by moral connotations, stigma, confidentiality, and privacy concerns, so they are unwilling to undergo CT screening. Home-based CT testing, which ensures confidentiality, may be an option to include this population in the screening strategies [26]. Finding a way to normalize and destigmatise chlamydia may also be another strategy to include this population [25, 27]. Future focus for asymptomatic patients, because their willingness to undergo routine CT screening was high, should be the acceptability of CT screening to providers if we want to promote routine CT screening intervention in clinics.

Our study provides evidence and implications for public health interventions on routine CT screening, and suggests that targeted interventions are urgently needed for particular sub-populations, including men, and those with lower education, shorter length of residency, lower monthly income, less awareness about the dangers of CT infection, and with STI-related symptoms.

We found 201 participants, accounting for 1.2% of the all participants, reported never having been tested and also reported that they had been diagnosed as having a chlamydia infection. There may be at least two possible reasons for this seemingly contradictory observation. First, syndromic diagnosis is still used by some doctors to simply make a patient diagnosed as having a chlamydia infection. Second, recall bias on testing or diagnosis may occur in some patients.

Several limitations exist in the present study. First, the representativeness of this study should be considered by using the convenience-sampling method to include participants. The first 15 eligible patients per day in each study site may be younger, as patients have to make an online appointment (mostly through an app in the smartphone) for an early visit. However, with the inclusion criteria of participants’ age (between 18 and 49 years) and a relatively large sample size (16,546 participants) across 6 of 10 city districts and 22 hospitals, the representativeness of our study could be reliable. The generalizability of this study should also be considered by using the convenience-sampling method, which recruited the first 15 eligible patients per day in each study site. In Shenzhen, participants who make an online appointment have priority to see a doctor, which may be different from other cities or countries with limited resources. Second, social desirability bias related to sexual and health behaviors may exist in the questionnaire survey. Third, as we did not collect data on those patients who rejected to participate in the survey, it is impossible to know the comparability of this group of patients with those who participated in the survey, resulting in the possibility of selection bias. Fourth, we did not provide information for participants about what type of annual CT testing they would take. This study provided a basic understanding of the willingness to undergo routine CT screening among hospital-based patients in China, and further research on more specific types of routine CT screening (e.g., free CT testing) is needed. Lastly, this study focused on willingness, and not behavioral intentions or actual behavior (e.g., acceptability of CT screening). Future studies are needed to find out how to translate the high willingness of routine CT screening to actual acceptability of CT screening. The associated factors that we found (e.g., gender, symptom status, awareness of the dangers of CT infection) may indicate an initial point for developing promising intervention strategies in this area.

## Conclusion

In summary, this study reported a high willingness to undergo routine CT screening among hospital-based patients in Shenzhen, which indicated that the CT screening programme should be scaled up in the above setting. Our findings suggested that gender, education level, length of residency, monthly income, CT knowledge, and STI-related symptoms are associated factors of willingness to undergo routine CT screening.

## Supplementary Information

**Additional file 1.** The questionnaire from the baseline study in the Shenzhen Gonorrhea and Chlamydia Intervention Programme; This file provides details of the questionnaire from the baseline study in the Shenzhen Gonorrhea and Chlamydia Intervention Programme.

## Data Availability

The datasets used and/or analysed during the current study are available from the corresponding author on reasonable request. Data request should be submitted to Dr. Yumao Cai (64165469@qq.com) who will review the data request with Ethical Review Committee of Shenzhen Center for Chronic Disease Control for approval.

## Abbreviations

AOR: Adjusted odds ratios
CI: Confidence interval
CT: Chlamydia trachomatis
NCSP: National Chlamydia Screening Programme
PID: Pelvic inflammatory disease
SD: Standard deviation
SGCIP: Shenzhen Gonorrhea and Chlamydia Intervention Programme
SPSS: Statistical Package for Social Sciences
STI: Sexually transmitted infection
VIF: Variance inflation factor
WHO: World Health Organization
