# Supplementary material for: Associated factors of willingness to undergo routine chlamydia trachomatis screening among hospital-based patients in Shenzhen, China: a cross-sectional study
Source: BMC Public Health. 2020 Nov 16;20:1720. doi: 10.1186/s12889-020-09828-6 (PMC7670722; doi:10.1186/s12889-020-09828-6)
Supplement: Supplementary file 1 — Additional file 1. The questionnaire from the baseline study in the Shenzhen Gonorrhea and Chlamydia Intervention Programme; This file provides details of the questionnaire from the baseline study in the Shenzhen Gonorrhea and Chlamydia Intervention Programme. [file 12889_2020_9828_MOESM1_ESM.docx]

**Questionnaire**

01 Department: ①Obstetrics and gynecology ②Urology ③Dermatology and venereology

02 Gender: ①Man ②Woman
03 Age (years): ______

04 Marital status: ①Single ②Married ③Divorced ④Widowed

05 Live separate and apart from your legal spouse or boyfriend/girlfriend? ①Yes ②No

06 Hukou: ①Shenzhen ②Others

07 Length of residency in Shenzhen: ①less than one year ②one year or more

08 Education: ①Primary school ②Middle school ③High school ④Technical secondary school ⑤Junior college or higher

09 Monthly income (RMB Yuan): ______;

10 Use health insurance: ①Yes ②No

11 Sexual orientation: ①Heterosexual ②Homosexual ③Bisexual
12 Have you ever been tested for Chlamydia trachomatis? ①Yes ②No
13 Have you ever been diagnosed with Chlamydia trachomatis infection? ①Yes ②No ③Forgot

14 Current symptoms of sexually transmitted infection: ①Yes ②No

15 Had a new sexual partner or multiple sex partners in last 3 months: ①Yes ②No

16 What do you know about genital Chlamydia trachoma infections? ①Never heard of it ②A kind of infectious disease ③A kind of genital tract infection ④A kind of sexually transmitted disease

17 What do you know about the dangers of genital chlamydia trachomatis infections on the human body? ①No dangers ②May affect sexual life ③May affect fertility ④Know nothing about it
18 Are you willing to undergo routine chlamydia trachomatis screening once a year? ①Yes ②No
